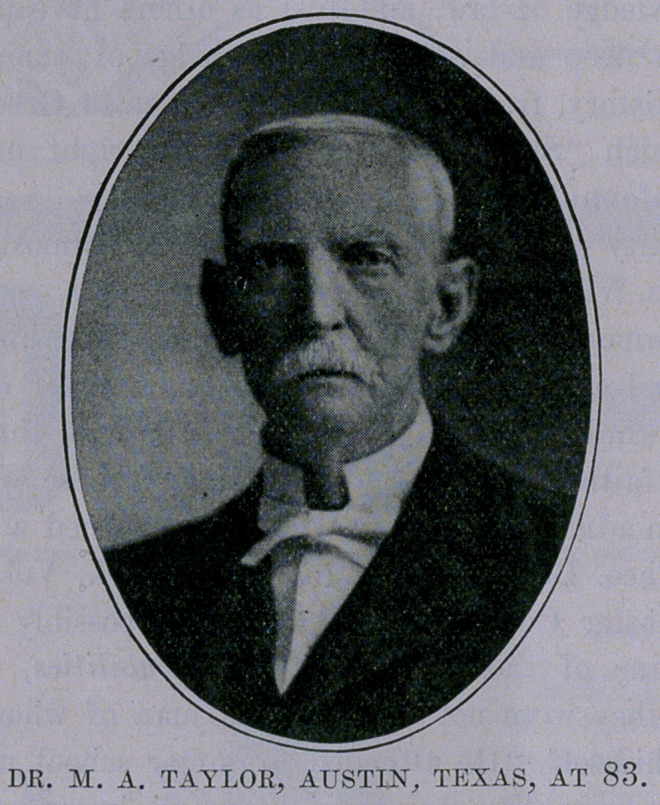# Dr. M. A. Taylor, Austin, Texas

**Published:** 1908-12

**Authors:** 


DR. M. A. TAYLOR, AUSTIN, TEXAS, AT 83.

---

Dr. M. A. Taylor is the pioneer physician of Austin and one of its early "landmarks." He was born in Columbus, Ohio, in 1825, and settled in Austin in 1852, a young graduate. He was the physician, personal friend and confidant of the illustrious Sam Houston, of whom he tells many interesting reminiscences. He has been in continuous practice in Austin fifty-six years, and is still active. He has accumulated great wealth, and has been a factor in the growth and development of the Capital City. At present he is a bank director and capitalist. His donations to the Church and to public enterprises have been large and numerous. During all this, more than half a century, he has had and retains the confidence, respect and esteem of the entire community. The present Mrs. Taylor—a beautiful and charming woman—was Bertha E. Moorman, of Michigan. She gave him a surprise party on his 83rd birthday (November 14th) by having a number of his oldest and closest friends to meet him at dinner, at which the writer had the honor to be present, and the conversation was like an echo from ante bellum times and full of interest.

## Figures and Tables

**Figure f1:**